# Exploring the Synergistic Action of Medium-Chain Triglycerides and Omega-3 Fatty Acids to Enhance Cellular Uptake and Anti-Inflammatory Responses

**DOI:** 10.3390/nu17111889

**Published:** 2025-05-31

**Authors:** Camila Kaminskas Fernandes Isern, Yao Chen, Roni Touboul, Benjamin Frank, Shuchen Hu, Chuchun L. Chang

**Affiliations:** 1 Institute of Human Nutrition, Vagelos College of Physicians and Surgeons, Columbia University, New York, NY 10032, USA; 2 Division of Gastroenterology, Hepatology, and Nutrition, Department of Pediatrics, Vagelos College of Physicians and Surgeons, Columbia University, New York, NY 10032, USA

**Keywords:** fatty acids, inflammation, immune response, MCT, omega-3

## Abstract

*Objectives:* Omega-3 fatty acids (*n*-3 FA) exhibit pro-healing and anti-inflammatory properties. Injectable lipid emulsions containing *n*-3 FA are being explored for the treatment of acute adverse conditions. Our previous studies demonstrated that a triglyceride (TG)-rich emulsion (TGRP) containing medium-chain TG (MCT) and *n*-3 TG (8:2 ratio) is rapidly cleared from the blood and efficiently taken up by organs. This study systematically examined the impact of varying MCT:*n*-3 ratios on cellular uptake and metabolic function in inflammatory processes. *Methods and results:* We measured the uptake of radio-labeled TGRP, comprising pure MCT, *n*-3, or mixed at selected ratios (8:2, 6:4, 2:8), both in vitro and in vivo. Murine macrophages with MCT:*n*-3 (6:4 or 2:8) had a 2-fold higher TG uptake. IV-injected mixed TGRP also enhanced blood clearance and organ uptake. *n*-3 TGRP reduced LPS-induced pro-inflammatory cytokines (TNF-α, IL-1, IL-6) in a dose-dependent manner. The 8:2 ratio enhanced mitochondrial respiration and glycolytic capacity in macrophages. Pro-inflammatory lipids decreased with MCT:*n*-3 (2:8) and pure *n*-3 TGRP. Bolus injections of *n*-3 TGRP with MCT lowered LPS-induced IL-6 in plasma and tissues. *Conclusions:* MCT and *n*-3 FA support metabolic activity and exhibit anti-inflammatory effects, suggesting that optimizing their ratio may enhance the therapeutic effects of emulsions for inflammatory conditions.

## 1. Introduction

Systemic inflammatory responses, often initiated by traumatic tissue injury, surgery, and infection, can result in cell death and organ damage [1,2,3]. Acute and ongoing inflammatory processes trigger the release of pro-inflammatory cytokines and chemokines that affect cellular signaling at the site of local inflammation. When pro-inflammatory mediators enter the systemic circulation, inflammation occurs in distal tissues or organs [4]. The rapid shifts in immune-cell recruitment alter tissue metabolism, and resultant changes in energy demand and supply can cause metabolic acidosis and hypoxia, leading to cell death [1,2,3]. Current anti-inflammatory treatments, like NSAIDs and monoclonal antibodies, often prove ineffective or have serious side effects, highlighting the need for a better understanding and management of inflammation to improve clinical outcomes [5,6,7,8].

The preservation of cell viability and tissue homeostasis during inflammation is linked to omega-3 (*n*-3) fatty acids (FA), particularly eicosapentaenoic acid (EPA) and docosahexaenoic acid (DHA), which are major structural and functional components of cells and cell membranes [9,10,11,12,13]. Bioactive *n*-3 FA alter membrane microstructures, modulate NF-κB- or PPAR-associated transcriptional regulation of inflammatory molecules, and catalyze generation of anti-inflammatory *n*-3-derived lipid mediators, such as specialized pro-resolving mediators (SPM, resolvins and protectins) [14,15] or other oxygenated metabolites that actively ameliorate inflammation [9,13,16,17,18,19]. However, the mechanisms underlying the enrichment of cells with *n*-3 triglycerides (TG) or FA and *n*-3 FA-associated amelioration of adverse inflammatory events remain unclear. Compared to dietary intake of fish or supplementation of *n*-3-rich FO, IV infusion of *n*-3-rich TGRP accelerates the incorporation of *n*-3 TG-derived FA into cells, exerting their anti-inflammatory and other beneficial effects [20]. This includes improved gas exchange and shortened length of hospital stay in critically ill patients [21]. Furthermore, recent data showed that acute bolus injection of emulsions rich in *n*-3 TG to mouse models protects against brain and cardiac tissue damage induced by cerebral hypoxic/ischemic (H/I) injury (stroke) [22], myocardial infarction [23], and spinal cord injury [24,25]. However, emulsions containing ~50% of their FA as EPA and DHA are very slowly hydrolyzed in vitro by lipoprotein lipase (LpL) and hepatic lipase, and *n*-3 FA contribute proportionally less than other FA to the total amount of FA released [26].

Medium-chain (MC) TGs (MCT), in contrast, are hydrolyzed much faster and may provide potential benefits for IV injection [27,28]. Accordingly, they have been combined with *n*-3 TG in parenteral nutrition (PN) [29] due to their physical and chemical properties [30,31]. MCTs are smaller in molecular weight and have shorter acyl tail lengths (6–12 carbons), which increase their solubility in membranes and may promote fluidity in cell membranes [29]. MCTs also travel more freely in circulation, desorb rapidly from membranes, diffuse quickly through cells, and are easily oxidized without requiring the carnitine transport system for mitochondrial entry [30,32]. They promote the hepatic production of ketones, which can fuel the brain [33] and the heart [34] when glucose is in short supply or unavailable due to insulin resistance, starvation, or critically ill conditions. We previously reported that emulsions with a combination of MCT with FO *n*-3 TG (MCT:FO = 8:2, wt/wt) were hydrolyzed more efficiently than emulsions containing either pure *n*-3 TG or combined MCT/long-chain TG (LCT)/*n*-3 TG (MCT:LCT:FO = 5:4:1, wt/wt/wt) [35], which leads to the formation of the remnant particles. We speculate that this enhanced lipolysis generates smaller remnant particles, which are more readily taken up by target cells in adjacent tissues. Moreover, the rapid release and oxidation of MC FA may spare *n*-3 FA from immediate β-oxidation, thereby preserving them for incorporation into membranes and availability for downstream anti-inflammatory signaling. Supporting this model, human studies have shown that bolus administration of MCT/FO (8:2) emulsions rapidly (<1 h) enriched cell membranes of white blood cells and platelets with *n*-3 FA [36], while animal studies have extended this enrichment in target metabolic organs, such as the liver [37,38].

FA β-oxidation in mitochondria serves as a primary pathway for supplying energy. MC FA can enter the mitochondrial matrix directly through passive diffusion, independently of CPT-1 at the outer mitochondrial membrane. MCT has been shown to provide an additional energy source for macrophages and potentially other immune cells to combat inflammation, possibly inducing reprogramming of macrophages from M1 status to a more M2-like status through a shift towards increased mitochondrial respiration [39]. Nonetheless, the effects of *n*-3 FA on metabolic activity have also been reported, particularly concerning their anti-inflammatory functions and their influence on the production of antioxidant enzymes, ROS, and oxidative stress in mitochondria [40,41,42,43]. However, how long-chain *n*-3 FAs are processed by mitochondrial β-oxidation in the presence of MCT has not been well-defined. Metabolic properties of MCT/FO emulsions have been investigated in cell culture models [35] as well as different animal models [44,45], including rat models with *n*-3 FA-depleted diets [46]; however, how their composition influences immune metabolism remains unclear. Although model TGRP metabolism is likely to follow pathways similarly to those of chylomicrons, which involve intravascular lipolysis of TG by LpL, releasing free FA (FFA) for uptake by adjacent tissues, TGRP remnant particles can also be taken up by tissues [29]. However, the metabolism of constituent FA can be affected by the TG composition of the emulsions [47,48]. Research on the inflammatory outcomes of mixed emulsions is still limited in the literature. To date, no study has systematically assessed how varying ratios of MCT and *n*-3 triglycerides within injectable emulsions affect both metabolic reprogramming and inflammatory outcomes in immune cells. Despite growing interest in the immunometabolic effects of FA, understanding how different FA profiles specifically impact metabolic and bioenergetic responses during inflammation remains limited. For example, in sepsis—a severe form of acute inflammation—mitochondrial dysfunction, elevated ROS production, and mitophagy are well-documented features [49]. We employed LPS-induced inflammation as a model of early-phase systemic immune activation. We compared TGRP with selected MCT:*n*-3 ratios to identify formulations that enhance uptake, metabolic preservation, and immune responses during acute inflammation. This work establishes a novel framework for ratio-based lipid emulsion design with therapeutic potential.

## 2. Methods

### 2.1. Materials

MCT, tri-DHA, tri-EPA, and cholesteryl oleate were obtained from NuChek Prep Inc. (Elysian, MN, USA). Egg yolk phosphatidylcholine was purchased from Avanti Polar Lipids, Inc. (Alabaster, AL, USA). Fatty acid-free bovine serum albumin (BSA), Dulbecco’s modified Eagle’s medium (DMEM), heparin sodium, and lactoferrin were obtained from Sigma-Aldrich (St. Louis, MO, USA). Sodium chlorate (NaClO_3_) was purchased from Fischer Scientific (Hampton, NH, USA). Penicillin, streptomycin, glutamine, and fetal bovine serum) FBS) were purchased from Gibco Invitrogen (Carlsbad, CA, USA). [Cholesteryl-1,2-^3^H(N)]-cholesteryl hexadecyl ether ([^3^H]CE) and scintillation fluid (Ultima Gold scintillation fluid) were purchased from PerkinElmer (Shelton, CT, USA).

### 2.2. Animal and Cell Models

Wild-type C57BL/6 male mice (~25 g) were purchased from the Jackson Laboratory (Bar Harbor, ME, USA). LpL-knockout mice expressing LpL exclusively in muscle (MCKL0) were generated by breeding transgenic mice that carried the muscle-specific creatine kinase (MCK) promoter driving a human LpL minigene onto the LpL knockout background. All mice were on a C57BL/6 background, as previously described [50,51]. All animals were housed in the Animal Facilities at Columbia University in a 12 h light/12 h dark cycle at room temperature. All animal procedures were approved by the Institutional Animal Care and Use Committee (IACUC) at Columbia University Irving Medical Center (AABG6550). Sample sizes were determined based on prior analyses and literature findings to ensure statistical rigor while minimizing animal use. Animals were randomly assigned to treatment groups, and outcome assessments were performed by blinded investigators when applicable.

J774.A2 murine macrophages were grown in DMEM growth medium containing 10% FBS and 1% penicillin/streptomycin at 37 °C for experimental use. This cell line was utilized because of its representative biological basis of the innate immune response under inflammatory circumstances, as previously described by Jung et al. [52].

### 2.3. Preparation and Characterization of Model TGRP

To establish the best ratio of different TG compositions for cellular uptake and optimization, we prepared TGRP comprising MCT and *n*-3 TG in different weight ratios by our reported methods [27,53,54]. To address potential concerns regarding the incremental or minor alterations in MCT/*n*-3 ratios, we selected the specific ratios of MCT and *n*-3 TG that allowed us to evaluate optimal cellular uptake. Table 1 lists different compositions of TGRP containing MCT (C8 trioctanoate), pure *n*-3 TG containing both EPA (C20:5) and DHA (C22:6) (ratio 1:1), or the combination of MCT and *n*-3 in different ratios (wt/wt). We used TGRP similar in size to chylomicrons that contain TG and phospholipids (PL) at the ratio of 8:1 (wt/wt), which were mixed and prepared by sonication using a sonifier disruptor (Branson Scientific, Inc., Danbury, CT, USA) [27,55,56]. TGRPs were made with a total TG content of 10 g/100 mL emulsion (10% by weight). Lab-made TGRPs were first characterized by visual inspection for creaming coalescence, oil separation, or color changes. TG and PL concentrations were characterized using colorimetric assays (Wako Chemicals USA Inc., Richmond, VA, USA) according to the manufacturer’s instructions. TGRP droplet size, stability, and particle distribution were examined using dynamic light scattering Zetasizer (Malvern Zetasizer Nano ZS, Malvern Panalytical, Malvern, UK) at the Columbia University Nano Initiative [57]. The charge of TGRP droplets [58] (zeta-potential), which has a major impact on TGRP globule size and stability, was determined by a Zetasizer [58,59]. The size measurements demonstrate that peaks of the TGRP are averaged at size < 200 nm. The zeta-potentials of the lab-made TGRPs are, in general, close to −30 mV, exhibiting desired narrow and high peaks (Table 1).

### 2.4. Preparation of Radiolabeled TGRP

To measure the cellular uptake of TGRP in vitro and in vivo, TGRPs were labeled with a trace amount of a non-metabolizable radioactive marker, [^3^H]CE. The introduction of [^3^H]CE did not alter the physical or chemical properties of TGRPs during the radiolabeling procedure [60]. [^3^H]CE was incorporated into the emulsion core by sonication. Radiolabeling efficiency was characterized for each batch by differential centrifugation methods as previously described to ensure that >90% of [^3^H]CE was recovered in the TG-rich section of the emulsion [61].

### 2.5. Cell TG Uptake/Mass Assays

[^3^H]CE-labeled TGRP was used to trace TG uptake and deposition in J774.A2 macrophages as previously described [35,62]. Cellular TG mass approximately equals TG deposition since basal cellular TG levels are minimally low. Cellular utilization is defined as the ratio of TG uptake/TG mass, as previously described [35,62].

In a similar experimental setting, J774.A.2 macrophages were incubated with various TGRPs for up to 4 h. Changes in lipid species in whole cell lysates, including TG, PL, cholesterol, cholesteryl esters (CE), and sphingolipids, were extracted and analyzed using an LC-MS/MS platform at Columbia Biomarker Core Laboratory [63]. Briefly, lipid extracts were prepared from cell lysates using a chloroform–methanol extraction method and spiked with appropriate internal standards and analyzed on a platform comprising Agilent 1260 Infinity HPLC integrated to Agilent 6490A QQQ mass spectrometer controlled by Masshunter v 7.0 (Agilent Technologies, Santa Clara, CA, USA) as described before [64]. Tree panels, scanning for either positive lipids, negative lipids or neutral lipids (under positive mode), were analyzed. Equal amounts of internal standards with known concentrations were spiked into each extract. Each standard was later used to calculate the concentrations of corresponding lipid classes by first calculating the ratio between the measured intensities of a lipid species and that of the corresponding internal standard multiplied by the known concentration of the internal standard. Lipid levels for each sample were calculated as nmol normalized for protein concentration.

### 2.6. In Vitro Characterization of TGRP Cell Uptake and Mass at Major Pathway Checkpoints

Based on the previous incubation protocol, the cells were treated with 1% BSA incubation media with [^3^H] CE-labeled TGRP in the presence or absence of competitive inhibitors to establish TGRP uptake pathways. We used lactoferrin to determine if the classical LDL receptor or apoE-mediated pathways would regulate TGRP uptake. The cells were incubated with 1mM of lactoferrin and a specific TGRP at 37 °C for 4 h [44]. The addition of apoE was used to validate the apoE-receptor-mediated uptake of TGRP (10 μg apoE/200 μg TG). For evaluating the cell proteoglycan as a non-classical pathway for TGRP uptake, the cells were pre-incubated with 50 mM of NaClO_3_ for 24 h or 4 µg/mL of pronase for 1 h to inhibit proteoglycan sulfation or to remove cell surface proteins, respectively [60,65], followed by incubation with specific TGRP. The particle cellular internalization of TGRP was measured as described [66].

### 2.7. Measurement of Metabolic Bioenergetics

Specific mitochondrial function parameters in live cells were measured by the Agilent Seahorse Xfe24 Extracellular Flux Analyzer (Agilent Technologies) [67]. The cultured cells were collected after incubation with specific TGRP treatments from 6-well plates. Cell counting was completed using a Neubauer chamber (Sigma-Aldrich, St. Louis, MO, USA). to be used for the calculation of the desired final concentration of 8.5 × 10^4^ cells per well. The cells were seeded onto Xfe24 24-well culture plate and centrifuged at 5000× *g* for 5 min prior to a 1 h incubation in a 37 °C non-CO_2_ incubator to allow cells to adhere. Culture media was prepared with pre-warmed Seahorse XF DMEM, with final concentrations of 1 mM pyruvate, 2 mM L-glutamine, and 10 mM glucose. Port solutions as drug injections for each Seahorse XF assay were prepared according to the manufacturer’s instructions. Oxygen consumption (OCR, pmol O_2_/min) and extracellular acidification rates (ECAR, mph/min) were analyzed using Mito Stress Test and Glycolysis Stress Test assays. Oligomycin (1 µM), carbonyl cyanide-4 [trifluoromethoxy] phenylhydrazone (FCCP; 1.5 µM), and rotenone/antimycin A mixture (0.5 µM) were used for measurements of mitochondrial respiration and ATP production. Glucose (10 mM), oligomycin (1 µM), and 2-deoxyglucose (2-DAG; 50 mM) were prepared according to the manufacturer’s guidelines for measurement of glycolytic flux and capacity. Specified volumes of each port solution were added to designated ports as per assay design.

After running the Seahorse XF assay, the cells were washed with PBS, lysed using Agilent recommended lysis buffer recipe (10 µM Tris-HCL, pH 7.4, 0.1% Triton ×100), allowed to rock in cold room for 30 min, and extracted by scraping. The supernatant was collected after centrifugation at 12,000 rpm for protein measurements using Bio-Rad DC (detergent compatible) protein assay (Bio-Rad Laboratories, Inc., Hercules, CA, USA) according to manufacturer’s instructions.

To induce inflammatory responses, J774.A2 cells were pretreated with emulsions at a concentration of 100 µg TG/mL for 2 h, followed by LPS treatment (1 µg/mL) mixed with 1% BSA growth medium and emulsion mixture for 18 h. The cells were then washed and plated onto Seahorse Extracellular Flux Analyzer microplates for metabolic analysis as outlined above.

### 2.8. Quantitative Real-Time PCR

To assess cytokine production, total RNA from cells was isolated using TRIzol reagent (Thermo Fisher Scientific, Waltham, MA, USA). Single-strand cDNAs were synthesized from RNA samples through reverse transcription (iScript, Bio-Rad, Hercules, CA, USA). Quantitative real-time PCR was completed using an iCycler (Bio-Rad) with SYBR Green PCR master kit (Applied Biosystems, Foster City, CA, USA). The selected gene sequences included 18S ribosomal RNA as the housekeeping gene for control, as well as pro-inflammatory markers IL-6, IL-1β, and TNF-α, to determine cytokine level changes with different emulsion conditions. Calculation of the relative levels of mRNA of each selected cytokine was performed by comparing the threshold crossing (Ct) of each sample after normalization to the control gene (ΔCt), using the formula 2^−ΔΔCt^.

### 2.9. Measurement of Mitochondrial Function

To measure specific mitochondrial function parameters in live cells without isolating mitochondria in real time, the Agilent Seahorse XF Plasma Membrane Permeabilizer was utilized [68]. The normalized OCR after sequential injection of the specific substrates and inhibitors in the permeabilized cells was used to calculate the activity of different components of the respiratory chain [69]. The complex I activity was determined by the reduction in OCR following the injection of rotenone, which inhibits complex I and halts NADH-linked respiration; the complex II activity was determined by the increase in OCR following the injection of succinate that drives respiration from electrons fed directly into the ubiquinone pool, and the Complex IV activity was determined by the increase in OCR following the injection of ascorbate/TMPD that deliver electrons to cytochrome C oxidase. The antimycin in Port C was injected to inhibit complex III, following the administration of rotenone and succinate, which allows ascorbate/TMPD at Port D to bypass the block at complex III.

### 2.10. Quantification of Inflammatory Markers by ELISA In Vivo

To evaluate the potential anti-inflammatory effects of *n*-3 containing TGRP in vivo, we measured the levels of C-reactive protein (CRP) and IL-6 in the plasma and metabolic tissue homogenates (liver and spleen) of mice following various doses of LPS injections by enzyme-linked immunosorbent assay (ELISA) using commercially available kits (CRP ELISA kits from Invitrogen (Carlsbad, CA, USA) and IL-6 ELISA kits from R&D Systems (Minneapolis, MN, USA) [70,71,72,73,74,75]. Our validation data indicate that, with a reduction in WBC and platelets, specific immune cell subsets involved in the innate immune response, notably monocytes and neutrophils, increased in mice 24 h post-LPS stimulation at doses ranging from 2 to 8 mg/kg BW (Supplemental Figure S1A). We selected a dosage of 2 mg LPS/kg BW for further analyses, as it induced comparable effects on increasing WBC subsets and the key plasma inflammatory indicator CRP levels determined by ELISA when compared to the higher doses (4 or 8 mg LPS) (Supplemental Figure S1B). Consistent with previous reports, this dose did not cause mortality [75]. Plasma CRP and IL-6 sharply increased between 4 and 8 h post-LPS. While IL-6 concentrations gradually declined after 4 h, CRP concentrations remained high at 24 h after the LPS injection (Supplemental Figure S1C). With the selected LPS dosing regime, the mice were administered various TGRPs before or after the LPS challenge. Plasma was collected post-injection via cardiac puncture while liver and spleen tissues were excised, weighed, and homogenized in lysis buffer containing protease inhibitors. The homogenates were centrifuged, and the supernatants were used for ELISA according to the manufacturer’s protocols. For both CRP and IL-6 assays, samples and standards were added to antibody-precoated plates, incubated, and then probed with detection antibodies, followed by substrate addition to develop the colorimetric reaction measured at a specific wavelength.

### 2.11. Statistical Analyses

The values were expressed as mean ± SEM. The statistical differences between group means were assessed by Student’s *t*-tests to compare endpoints or one-way ANOVA with post hoc tests to evaluate potential interactions between groups. All statistical tests assumed a 95% confidence level of a normal distribution. The statistical significance was determined at the level of *p*-values of <0.05. The statistical analyses were performed using GraphPad Prism (v.9.5.0).

## 3. Results

### 3.1. Cellular Uptake of MCT/n-3 TGRP In Vitro

**Figure 1:**
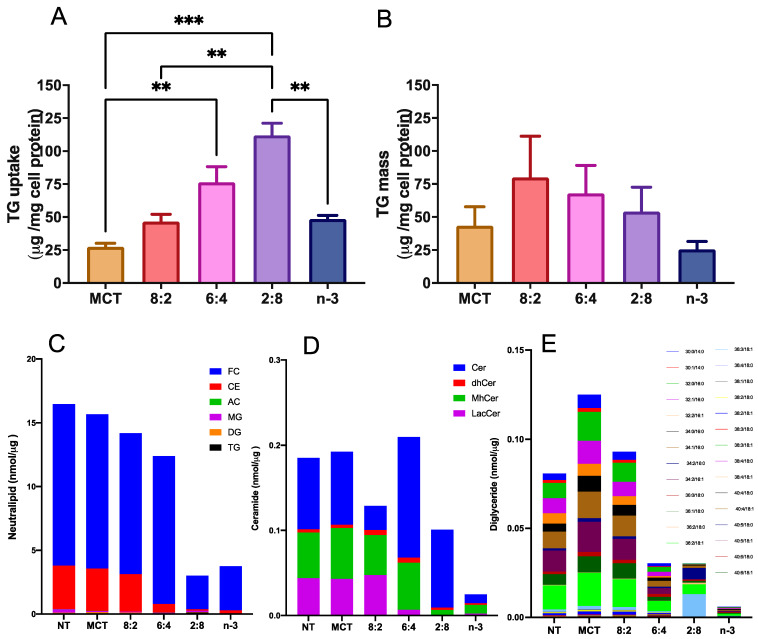
**n-3 TG combined with MCT increase cellular uptake in immune cells.** J774 macrophage cells were incubated with [^3^H]CE-labeled pure MCT, n-3 TGRP or TGRP in which n-3 TG was mixed with MCT (MCT:*n*-3 8:2, MCT:n-3 6:4, MCT:*n*-3 2:8) at 37 °C for 4 h (200 µg TG/mL). Cellular TG uptake (**A**) and cell TG mass deposition (**B**) were measured and analyzed as described in the Methods. Data were expressed as the mean ± SEM (n = 3–5) and denoted as ** *p* < 0.01, *** *p* < 0.001, utilizing one-way ANOVA followed by Tukey’s multiple comparisons. Cellular lipids were extracted from 3 replicate samples (~3 million cells) for the measurement of neutral lipids, including free cholesterol (FC), cholesterol ester (CE), acyl carnitines (AC) and glycerides (MG, DG and TG) (**C**), ceramide species, including ceramides (Cer), dihydroceramides (dhCer), monohydroceramides (mhCer) and lactosylceramides (LacCer) (**D**), and diglycerides (DG) with different chain lengths (**E**) through lipidomic analysis.

To assess the impact of varying MCT to *n*-3 ratios on cellular TG uptake and deposition, TGRPs were labeled with a trace quantity of the non-metabolizable marker [^3^H]CE, as outlined in our previous studies [35,76]. In initial dose–response and time-course experiments, we evaluated TG uptake and TG mass deposition in cultured immune effector cells-macrophages treated with TGRP. We found a significant slope (R^2^ > 0.90, *p* < 0.05) for all TGRPs within the TG dose of 200 µg/mL during a 4 h incubation period. Using a similar treatment regime, our results show that macrophage cells incubated with MCT/*n*-3 6:4 and 2:8 TGRP had the highest TG uptake (Figure 1A) compared to cells receiving other emulsions. However, lower TG mass deposition was observed in cells treated with MCT/*n*-3 2:8, comparable to those treated with lower uptake groups, such as pure *n*-3 or MCT TGRP (Figure 1B). Similar trends were observed in changes in several major lipid species, especially those that are key in mediating pro-inflammatory responses, such as ceramides (Cer) and diglycerides (DG), after increasing *n*-3 content in pool whole cell lysates after a 4 h incubation (Figure 1C–E) by lipidomic. When we calculated the efficiency of TG utilization defined by dividing TG mass by the uptake, we found that MCT TGRP was least utilized in the treated cells compared to cells treated with other TGRP, while the 2:8 or *n*-3 TGRP-treated cells had higher rates of TG utilization when compared to other groups (Supplemental Figure S2).

### 3.2. Blood Clearance Kinetics of MCT/n-3 TGRP In Vivo

**Figure 2:**
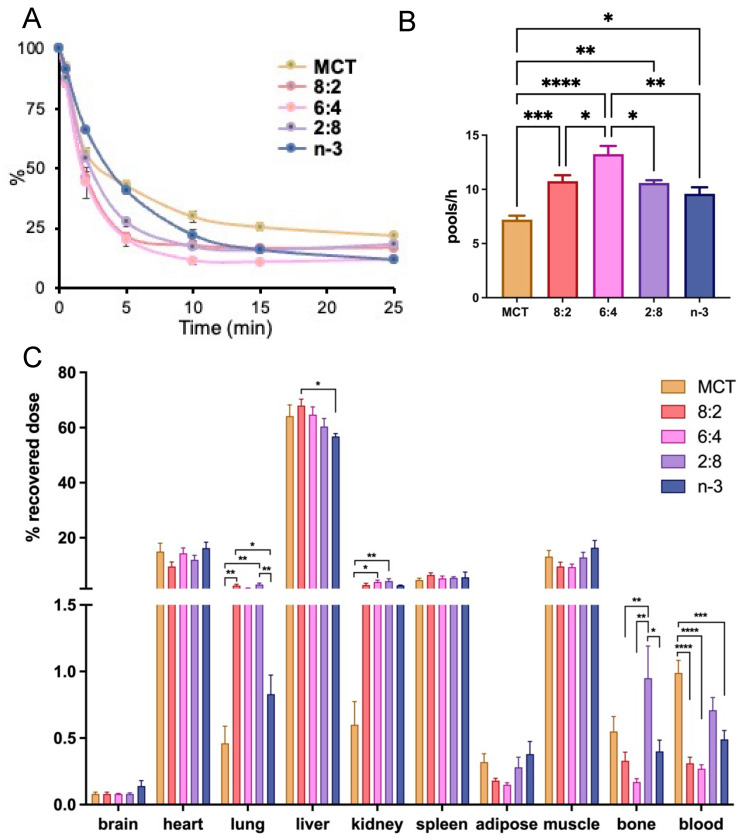
**Mixing n-3 TG with MCT accelerates blood clearance and hepatic lipid uptake.** MCT and *n*-3 TGRP or TGRP in which n-3 TG was mixed with MCT (MCT:*n*-3 8:2, MCT:*n*-3 6:4, MCT:*n*-3 2:8) were radiolabeled with [^3^H]CE and intravenously injected into C57BL/6J mice at the dose of 0.016 mg TG/g BW. (**A**) Blood clearance of the injected TGRP was measured at 0.5, 2, 5, 10, 15, and 25 min post-injection. (**B**) Fractional catabolic rate (FCR) was calculated based on the of first-order linear kinetics during the first 10 min. (**C**) Organ uptakes of radiolabeled TGRP were expressed as a percent of the sum of the radioactivity of all organs assayed at the end of the experiment. 42% and 15% of body weight was accounted for muscle mass and adipose tissue mass, respectively, as described in the Methods. Data were expressed as the mean ± SEM, n = 4–8, * *p* <0.05, ** *p* < 0.01, *** *p* < 0.001, **** *p* < 0.0001, using one-way ANOVA followed by Tukey’s multiple comparisons.

In our prior reports, we demonstrated that the blood clearance rate of the MCT:FO 8:2 emulsion in rodent models was faster than other emulsions with different TG contents, such as soybean LCT or emulsions comprising distinct ratios of MCT and FO, e.g., 5:4:1 (50% MCT, 40% LCT, and 10% FO, wt/wt/wt) [61]. Using a similar approach to obtain first-order kinetics with [^3^H]CE to trace emulsions containing different core TG compositions at a non-saturating dose of 0.016 mg TG/g BW, we observed accelerated blood clearance rates (Figure 2A) and fractional catabolic rates (FCR) (Figure 2B) in mice after IV injection of mixed MCT and *n*-3 TGRP compared to those injected with pure MCT or *n*-3 TGRP. Among the TGRP mixed with both MCT and *n*-3, the mice injected with the MCT:*n*-3 6:4 TGRP demonstrated the highest FCR.

We also investigated organ uptake in mice administered mixed MCT and *n*-3 TGRP compared to those injected with pure MCT or *n*-3 TGRP (Figure 2C). While similar tissue uptake and distribution patterns were observed among mice that received IV injections of TGRP, with over 60% of particles taken up by the liver, the mice injected with mixed MCT/*n*-3 8:2 and 6:4 TGRP demonstrated increased liver uptake attributed to accelerated blood clearance rates and FCR. On the other hand, mixed MCT and *n*-3 TGRP at 2:8 consistently showed a greater proportion of non-hepatic tissue uptake, including bone, similar to that in the mice injected with pure MCT (Figure 2C).

### 3.3. Cellular Uptake Pathways for Internalizing MCT/N-3 TGRP In Vitro and In Vivo

**Figure 3:**
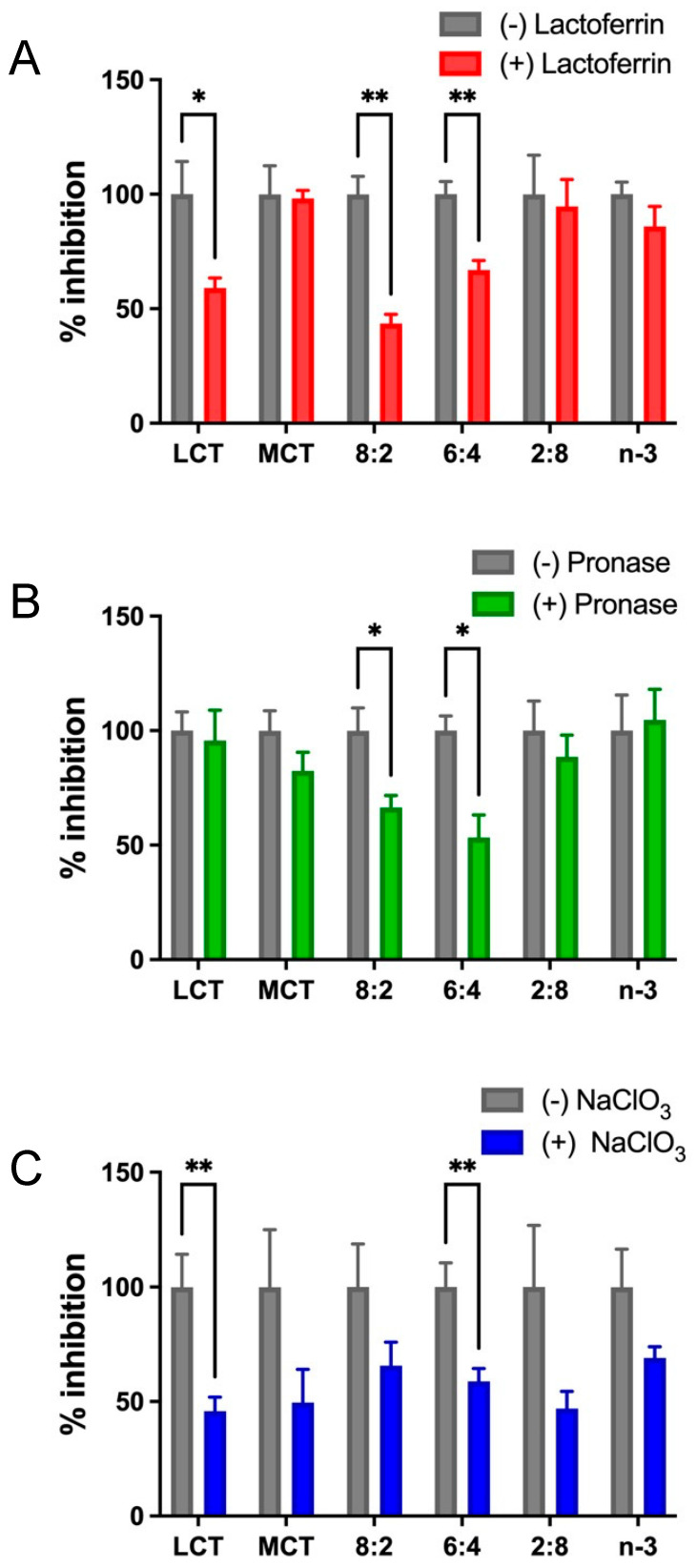
**Mixed MCT and n-3 TGRP utilize both receptor-mediated and cell surface protein-directed uptake pathways.** J774 cells were incubated with [^3^H]CE-labeled LCT, MCT, and *n*-3 TGRP or TGRP in which *n*-3 TG was mixed with MCT (MCT:*n*-3 8:2, MCT:*n*-3 6:4, MCT:*n*-3 2:8) at 37 °C for 4 h (200 µg TG/mL each emulsion) in the absence or presence with (**A**) lactoferrin (1 mM, n = 3); (**B**) pronase (4 µg/mL, n = 3); (**C**) sodium chlorate (NaClO3, 50 mM, n = 6) as described in the Methods. Data were expressed as the mean ± SEM, * *p* <0.05, ** *p* < 0.01 with the multiple unpaired *t*-test.

The uptake or transfer of model TGRP can be mediated by membrane-bound receptors (LDL receptor, apoE), membrane proteins (HSPG, FA transporter), receptor components such as LDL-R-associated protein (LRP), or LpL. We used well-characterized inhibitors at major catabolic uptake checkpoints to define the cellular pathways of MCT:*n*-3 TGRP at ratios of 8:2, 6:4, or 2:8 in innate immune cell and mouse models. Lactoferrin is known to inhibit the clearance of remnant lipoproteins from the plasma and competes with the cell-surface binding of apolipoprotein (apo)E-enriched remnants. Additionally, lactoferrin also inhibits remnant binding and the uptake of TGRP by interacting with both heparan sulfate proteoglycans (HSPG) and LRP. As expected, the presence of lactoferrin decreased LCT uptake in macrophages by 45% [65,76]. Major reductions in cellular TG uptake or mass deposition were also observed in cells treated with MCT:*n*-3 8:2 and 6:4 TGRP with the addition of lactoferrin (Figure 3A). To examine whether the cell proteoglycans may serve as a non-classical mediator in the uptake pathway for MCT:*n*-3 emulsions, we used sodium chlorate (NaClO_3_) and pronase to inhibit the synthesis of proteoglycans and to remove cell-bound proteins, respectively. Pre-incubation with pronase reduced uptake mainly in cells treated with MCT:*n*-3 8:2 and 6:4 TGRP. While NaClO_3_ decreased cellular TG uptake in LCT-treated cells by 50% (*p* < 0.05), significant reductions in cellular TG uptake (*p* < 0.05) were also observed in cells incubated with MCT:*n*-3 6:4 TGRP in the presence of NaClO_3_ (Figure 3C). TG cell mass deposition for all tested TGRP with inhibitors displayed a similar trend (ns) to TG uptake.

**Figure 4:**
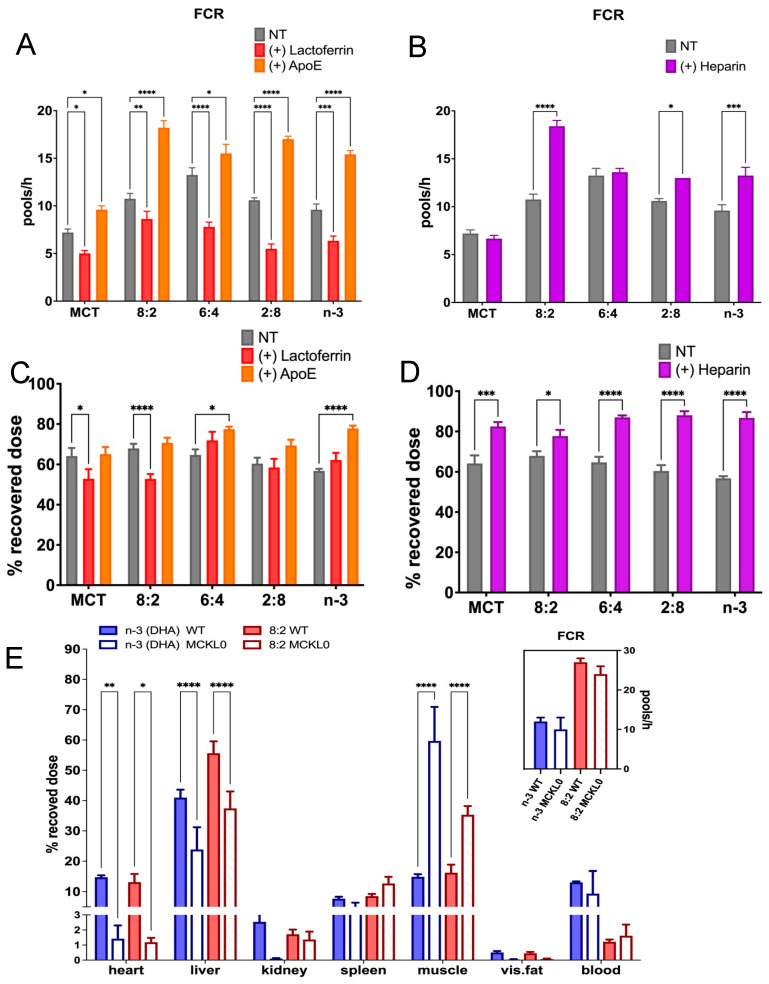
**Metabolic uptake pathways of MCT and n-3 TGRP in vivo.** C57BL/6J mice were intravenously injected with [^3^H]CE-labeled emulsions (0.016 mg TG/g BW) in the absence of the presence of (**A**) lactoferrin (0.07 mg/g BW), apoE, or (**B**) heparin (0.4 units/g BW) injected 1 min before TGRP injection. Fractional catabolic rates (FCR) were assessed as described in the Methods. (**C**) Hepatic uptake of TGRP in mice treated with lactoferrin and apoE. (**D**) Hepatic uptake of TGRP in mice treated with heparin. (**E**) Organ uptake of TGRP in LpL-knockout mice (insert: FCR). Data were expressed as Mean ± SEM, * *p* < 0.05, ** *p* < 0.01, *** *p* < 0.001, **** *p* < 0.0001, utilizing 2-way ANOVA followed by Dunnett’s multiple comparisons ((**A**–**D**), n = 4 or 5), or Tukey’s multiple comparisons ((**E**), n = 3–8).

To investigate whether lactoferrin acts as a significant inhibitor at TGRP metabolic checkpoints in vivo, we administered lactoferrin to mice prior to TGRP injection (Figure 4A). The dose of lactoferrin (17.5 mg/250 kg BW) was selected to achieve a plasma concentration of approximately 30 moles/liter. This concentration has been shown to inhibit up to 200 g of chylomicron remnant uptake that acquired ~100 nm of apoE, leading to a 50–70% reduction in liver uptake of the remnants [44,66,77]. Our results demonstrate that the pre-injection of lactoferrin resulted in slower blood clearance for all injected groups (as shown in Figure 4A). Conversely, the injection of apoE accelerated blood clearance rates, as measured by FCR. Changes in blood clearance rates were mainly attributed to the levels of hepatic uptake of pure MCT and MCT:*n*-3 8:2 TGRP (Figure 4C), while the reduction in mice injected with MCT:*n*-3 6:4, 2:8, and pure *n*-3 resulted from decreased uptake in non-hepatic tissues, such as the heart and muscle (Supplemental Figure S3A). The impacts of apoE-mediated uptake in tissues are varied, given the diverse TG composition within different TGRP and the dependence on the specific types of tissues where the uptake occurs.

We determined whether these differences in uptake could be attributed to the various TG hydrolysis rates in cells for these TGRPs [26]. In line with our previous findings, the injection of heparin enhanced plasma LpL lipolytic activity, accelerating blood clearance and the FCR of all tested TGRP, but had little to no effect on that of MCT or 6:4 in mice (Figure 4B). However, heparin significantly enhanced hepatic uptake across all injected groups (Figure 4D), while non-hepatic uptake decreased proportionally, as seen in the heart and adipose tissues (Supplemental Figure S3B). As a key lipolytic enzyme directing cellular uptake of lipids or lipoproteins at peripheral tissues, we found that LpL deficiency affected not only the hepatic uptake of chylomicron-sized TGRP, regardless of the TG composition, but also impacted other metabolic tissues, such as the heart and adipose tissues. It is worth noting that the expression of LpL in the muscle of MCKL0 mice is associated with increased uptake of injected TGRP (Figure 4E). However, as these LpL-KO mice were rescued by expressing LpL in muscle to preserve partial lipolytic activity, the FCR of various TGRPs was not affected in LpL-deficient mice compared to the control mice (Figure 4E, insert). From these findings, we speculate that regulating LpL expression and function in different tissues plays an essential role in maintaining a balance of lipid metabolism and energy homeostasis.

### 3.4. Mitochondrial Activity and Glycolytic Flux in Macrophages Treated with Different TGRP

**Figure 5:**
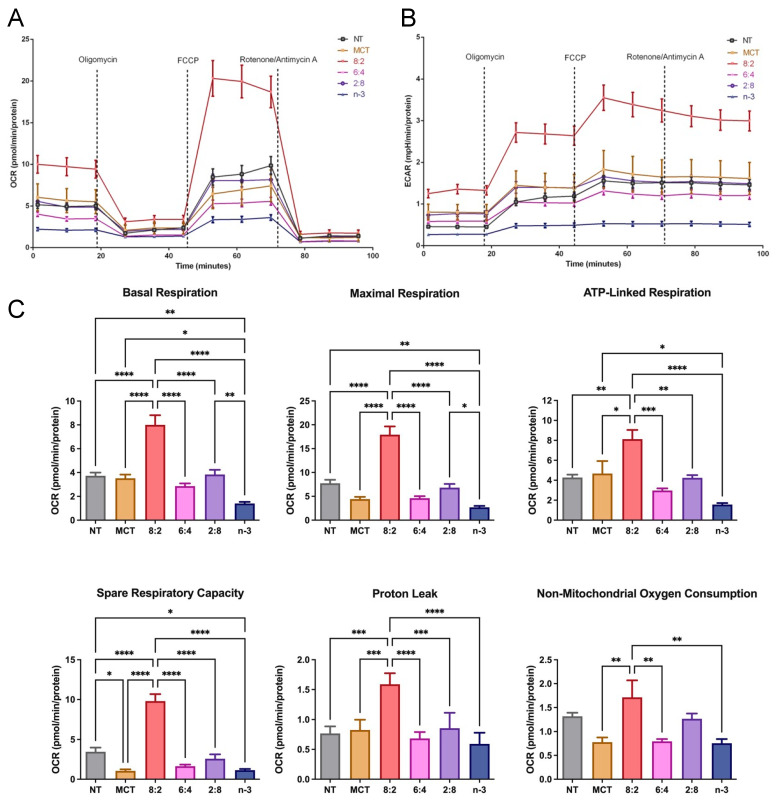
**Mitochondrial activity in macrophages treated with MCT and n-3 TGRP in vitro.** The (**A**) OCR and (**B**) ECAR were measured through Mito Stress Test using Seahorse Extracellular Flux Analyzer. J774 macrophage cells were injected with oligomycin, FCCP, and rotenone/antimycin A to evaluate the mitochondrial oxidative phosphorylation levels after no treatment or treatment with MCT, *n*-3 TGRP, or TGRP in which n-3 TG was mixed with MCT (MCT:*n*-3 8:2, MCT:*n*-3 6:4, MCT:n-3 2:8). (**C**) Mitochondrial parameters were calculated based on OCR from Mito Stress Test. Data were expressed as Mean ± SEM, n = 5, * *p* < 0.05, ** *p* < 0.01, *** *p* < 0.001, **** *p* < 0.0001, compared with NT group by one-way ANOVA with Tukey’s multiple comparisons.

Mitochondrial β-oxidation is a key energy-generating pathway. Medium-chain FAs enter the mitochondrial matrix via passive diffusion, bypassing CPT-1, whereas how long-chain *n*-3 FAs are oxidized in the presence of MCT remains unclear. We determined whether experimental MCT:*n*-3 TGRP at different levels affects FA metabolism in macrophage cells using the Seahorse analytical bioenergetics approach [67]. Our data show that the overall mitochondrial OCR was upregulated only by MCT:*n*-3 8:2 TGRP compared to that in the non-treated control cells or cells treated with other TGRP (Figure 5A). The glycolytic flux measured by ECAR was also significantly increased by 8:2 TGRP compared to the control (Figure 5B). Other TGRP, such as 6:4 and 2:8, displayed little or no change in OCR and ECAR compared to the control (Figure 5A,B). The analysis of specific mitochondrial function parameters shows that the 8:2 TGRP significantly increased basal and maximal respiration, ATP production, and spare respiratory capacity. On the other hand, pure *n*-3 TGRP caused a significant reduction in overall mitochondrial respiration and functional indicators (Figure 5C). Similar changes in non-mitochondrial oxygen consumption were observed in these macrophage cells.

**Figure 6:**
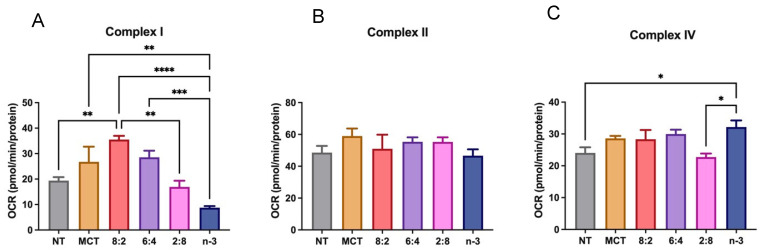
**Electron transport chain complex activity in macrophages treated with lipid emulsions.** J774 macrophage cells treated with MCT, *n*-3 TGRP or TGRP in which *n*-3 TG was mixed with MCT (MCT:*n*-3 8:2, MCT:*n*-3 6:4, MCT:*n*-3 2:8) were injected with rotenone, succinate, antimycin, ascorbate/TMPD to determine (**A**) complex I activity (**B**) complex II activity, and (**C**) complex IV activities, using Seahorse Extracellular Flux Analyzer. Data were expressed as Mean ± SEM, n = 5, * *p* < 0.05, ** *p* < 0.01, *** *p* < 0.001, **** *p* < 0.0001, analyzed by one-way ANOVA with Tukey’s multiple comparisons.

To unveil the underlying mechanism of the decreased OCR and ECAR mediated by *n*-3 TGRP treatment, we examined whether this is associated with the activity of major respiratory enzymes, such as complexes II, III, and IV, and the FA oxidation flux pathways [78] in model macrophage cells. Our results confirm that MCT:*n*-3 8:2 activated complex I activity. Importantly, increasing *n*-3 TG content suppressed complex I activity, which may lead to reduced ROS production. The cellular oxidative phosphorylation appeared preserved by the increased complex IV activity in cells treated with higher *n*-3 content. (Figure 6).

**Figure 7:**
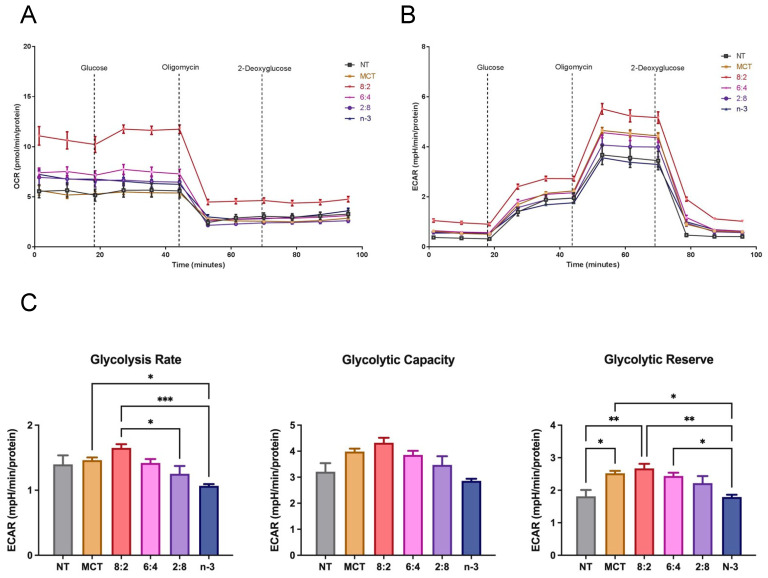
**Glycolytic activity in macrophages treated with MCT and *n*-3 TGRP in vitro.** The (**A**) OCR and (**B**) ECAR were measured through Glycolysis Stress Test using Seahorse Extracellular Flux Analyzer. J774 macrophage cells were injected with glucose, oligomycin and 2-DAG to evaluate glycolytic function after no treatment or treatment with MCT, *n*-3 TGRP or TGRP in which *n*-3 TG was mixed with MCT (MCT:*n*-3 8:2, MCT:*n*-3 6:4, MCT:*n*-3 2:8). (**C**) Glycolytic parameters were calculated based on ECAR data from Glycolysis Stress Test. Data were expressed as Mean ± SEM, n = 5, * *p* < 0.05, ** *p* < 0.01, *** *p* < 0.001, compared with NT group by one-way ANOVA followed by Tukey’s multiple comparisons.

We also evaluated glycolytic function in macrophage cells following treatment with different TGRPs (Figure 7). The MCT:*n*-3 8:2 TGRP increased OCR and ECAR compared to control (Figure 7A,B) in instigating glycolysis using a series of metabolic modulators. Pure MCT, 6:4, and 2:8 TGRP also increased ECAR, while pure *n*-3 TGRP caused a slight decline in ECAR. Overall, the glycolytic rate was mildly increased with 8:2 TGRP but not significantly compared to the control. Glycolytic capacity was increased after treatment with all TGRPs except for *n*-3 TGRP, with no significant differences observed. The glycolytic reserve was significantly higher in cells treated with 8:2 and 6:4 emulsions (*p* < 0.05), indicating greater flexibility and capacity to adapt by increasing glycolytic rates upon inhibiting mitochondrial respiration.

### 3.5. Mitochondrial Activity and Glycolytic Flux in Macrophages Stimulated with LPS

**Figure 8:**
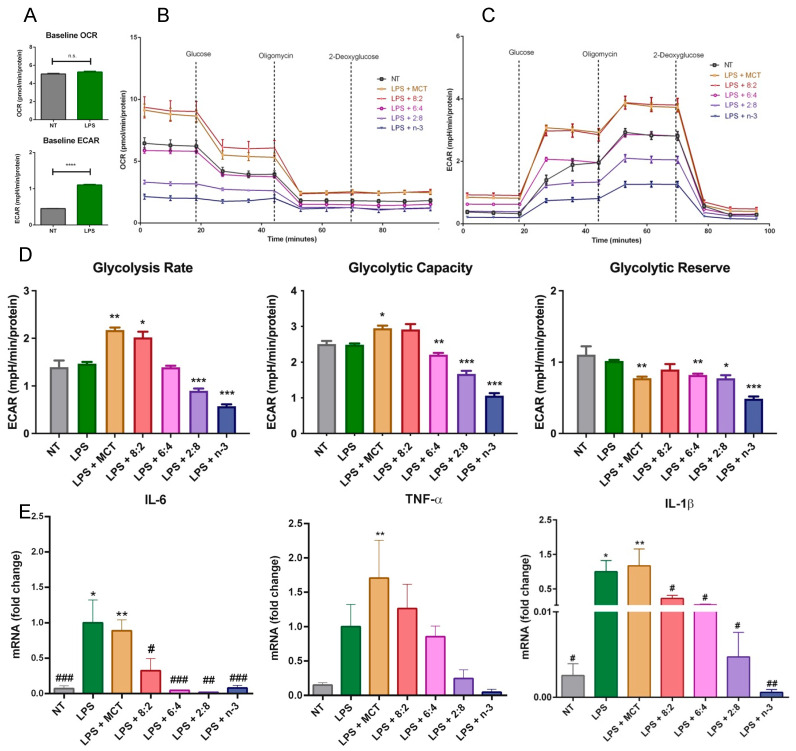
**Cellular metabolism in macrophages after inflammatory stimulation and treatment with MCT and n-3 TGRP in vitro.** (**A**) Baseline OCR and ECAR measurements were taken from non-stimulated J774 macrophages and those incubated with LPS (1 µg/mL for 24 h). **** *p* < 0.0001; Unpaired *t*-test results were used. The (**B**) OCR and (**C**) ECAR were assessed using the Glycolysis Stress Test and the Seahorse Extracellular Flux Analyzer. LPS-stimulated macrophage cells were subsequently injected with glucose, oligomycin, and 2-DAG to evaluate glycolytic function, either with no treatment or treatment with MCT, n-3 TGRP, or TGRP, where *n*-3 TG was mixed with MCT. (**D**) Glycolytic parameters were calculated based on ECAR data obtained from the Glycolysis Stress Test. * *p* < 0.05, ** *p* < 0.01, *** *p* < 0.001 when compared with the LPS group via one-way ANOVA. (**E**) The mRNA fold change of pro-inflammatory markers in J774 macrophage cells stimulated with LPS and treated with various TGRPs was assessed. n = 5; * *p* < 0.05, ** *p* < 0.01, compared with the NT group; # *p* < 0.05, ## *p* < 0.01, ### *p* < 0.001 compared with the LPS group. Analyzed using one-way ANOVA with Dunnett’s multiple comparisons.

The effects of *n*-3 FA on metabolic activity have been actively explored, especially in relation to their anti-inflammatory functions. We thus evaluated the effects of the combination of both MCT and *n*-3 TG on resident immune cell functional and metabolic activities in response to an immune stimulus LPS that has previously been shown to increase glycolysis rates to maintain energy production in macrophages, in part due to greater cytokine production [79,80,81]. LPS concentration and incubation time were validated based on the preliminary results for subsequent experiments in J774 macrophage cells, where LPS increased cytokine levels to the greatest extent at a concentration of 1 µg/mL after 24 h (Supplemental Figure S4). Our in vitro data show the expected increase in ECAR or glycolytic rate in cells after LPS stimulation (Figure 8A). MCT and 8:2 TGRP increased OCR and ECAR in LPS-treated macrophages. Decreased ECAR was associated with increasing *n*-3 content (Figure 8B). Analysis of specific glycolytic functions revealed that the rates of glycolysis and glycolytic capacities were increased following treatment with MCT and 8:2 TGRPs and significantly decreased with 2:8 and *n*-3 TGRPs. Conversely, the glycolytic capacity decreased in cells treated with 6:4, 2:8, and *n*-3 TGRPs (Figure 8D). The glycolytic reserve was significantly reduced in cells with all TGRP treatments except for 8:2 in response to LPS.

The results on mRNA expression of pro-inflammatory cytokines show that 3 h pre-treatment of MCT TGRP had little effect on influencing LPS-induced expression of pro-inflammatory TNF-α, IL-1β, and IL-6 (Figure 8E). By contrast, all *n*-3-containing TGRPs exhibited substantial inhibitory effects by suppressing the expression of these pro-inflammatory cytokines in a dose-dependent manner (Figure 8E) [43].

### 3.6. Anti-Inflammatory Action of TGRP in Response to LPS In Vivo

**Figure 9:**
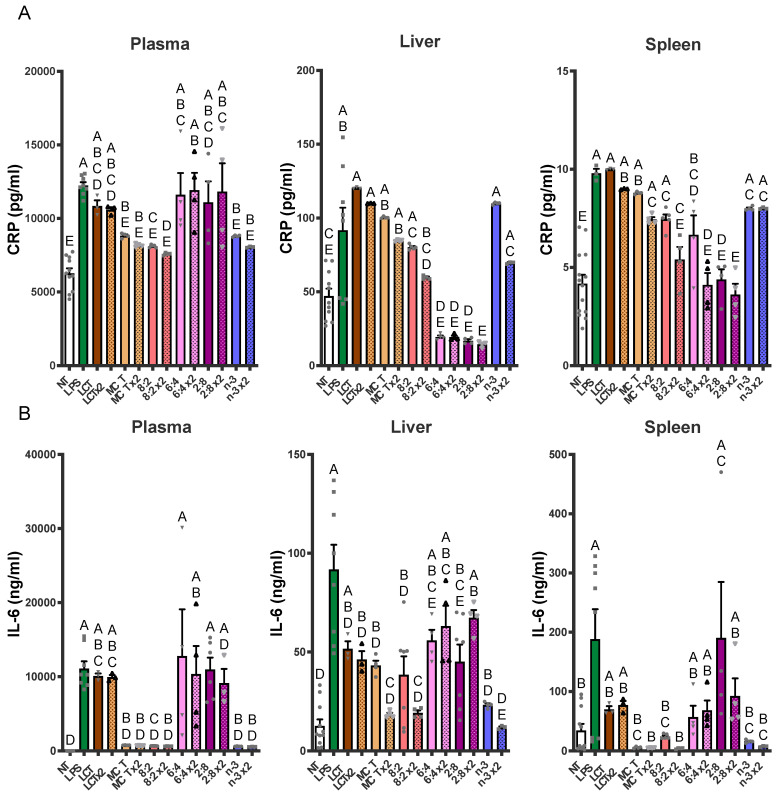
**Bolus n-3 TG injection attenuates LPS-induced pro-inflammatory marker levels in adult mice**. C57BL/6J mice were pre-injected with a non-saturating dose of *n*-3 TGRP (0.016 g TG/Kg BW) either 1 h before the LPS injection (filled bars) or through two separate injections (x2, patterned bars): 1 h before and another injection 1 h after the initial LPS administration. Plasma and tissue (liver and spleen) pro-inflammatory CRP (**A**) and IL-6 (**B**) levels were assayed by ELISA as described in the Methods. NT: saline-injected vehicle control; n-3: LPS + *n*-3 treated groups. Data were expressed as Mean ± SEM, n = 5. Bars labeled with different letters are significantly different from each other (*p* < 0.05), analyzed by one-way ANOVA followed by Tukey’s multiple comparisons.

We further examined the effects of combining MCT and *n*-3 TG on immune cell responses to LPS-induced acute inflammation in vivo [70,71,72,73,74,75]. Using the validated results on IL-6 and CRP as indicators for inflammatory response, we explored the therapeutic potential of experimental *n*-3 TGRP in mitigating acute LPS-mediated inflammatory responses in the mouse model. We administered the non-saturating dose of *n*-3 TGRP (0.016 g TG/kg BW) either once (1 h before LPS injection) or twice (1 h before and 1 h after LPS injection). Six hours post-LPS, levels of CRP and IL-6 were measured in plasma, liver, and spleen to assess systemic and tissue-specific inflammatory responses (Figure 9A,B). As expected, LPS injection induced an augmentation of pro-inflammatory markers, including CRP and IL-6, in plasma and the spleen, which serve as secondary reservoirs of immune cells, as measured by ELISA (Figure 9). This effect was also observed in the liver, a major metabolic tissue responsible for producing CRP and IL-6. Treatment with LCT exhibited no effect or may have exacerbated the LPS-stimulated production of CRP and IL-6.

In plasma, LPS-induced increases in CRP were notably reduced by dual doses of MCT, *n*-3 or MCT:*n*-3 at a 8:2 ratio (*p* < 0.05 vs. LPS). Single doses of these TGRP treatments also decreased CRP, but less effectively. IL-6 levels in plasma showed a dramatic increase following LPS but were strongly suppressed by single or dual doses of MCT, *n*-3 or 8:2 emulsions (*p* < 0.05). Although MCT:*n*-3 6:4 or 2:8 TGRP did not significantly affect plasma CRP or IL-6 levels, liver CRP levels decreased the most with these TGRP treatments (*p* < 0.05). IL-6 levels reflected those observed in plasma, with *n*-3 ×2 and 2:8 ×2 yielding the highest reductions, while other groups had minimal improvement.

In the spleen, CRP suppression was less pronounced, although 8:2 ×2, 6:4 ×2, and 2:8 ×2 still achieved reductions, indicating variable tissue-specific responsiveness (Figure 9A). A similar trend was observed for IL-6, with suppression being most pronounced in the MCT ×2, 8:2 ×2, and *n*-3 ×2 groups (Figure 9B). Collectively, these findings indicate that lipid emulsions rich in *n*-3 TG, especially when administered both before and after the LPS challenge, are highly effective in attenuating LPS-induced inflammation. Mixed *n*-3/MCT content and repeated dosing clearly enhanced the anti-inflammatory response, emphasizing the importance of formulation and treatment strategy in modulating acute inflammatory responses.

## 4. Discussion

A gap in knowledge persists regarding the various oil compositions of lipid emulsions related to energy production, mitochondrial function, delivery, and inflammatory outcomes. Over time, the applications of lipid emulsions have evolved from providing general nutrition provision through PN to offering individualized treatment for clinical diseases. Both MCT and FO:*n*-3 TG have been used as core TG components for IV lipid emulsions. However, only MCT:FO 8:2 emulsions were studied extensively, and this ratio has not been compared against other ratios [35]; there have not been any studies that tested MCT and pure *n*-3 mixed emulsions and their metabolic properties in experimental cell or animal models. This study demonstrates that MCT:*n*-3 TGRP in ratios of 6:4 and 2:8 provided the highest cellular TG uptake in cell and animal models. Our findings suggest that the accelerated rates of cellular uptake in cells or mice that received mixed MCT and *n*-3 TGRP might be due to their ability to utilize both receptor- and non-receptor-mediated uptake pathways, such as the cell surface proteoglycans as part of the non-classical TG uptake pathway for MCT:*n*-3 emulsions. At the same time, our study suggests that MCT:*n*-3 TGRP with a higher MCT ratio (8:2) is distinct in preserving metabolic activity in macrophages and rapidly attenuating adverse inflammatory response. Our novel findings demonstrate that the anti-inflammatory and metabolic effects of lipid emulsions are not only driven by the presence of MCT or *n*-3 TG alone but also depend on their specific ratio, with the 8:2 formulation providing multifaceted effects across various tissues.

In this study, we investigated whether the cellular uptake of model TGRP is mediated by membrane-bound receptors (e.g., LDL-R), membrane proteins (e.g., HSPG), or receptor components like LRP. In macrophages, MCT:*n*-3 TGRPs at 6:4 and 8:2 ratios exhibited apoE-dependent binding to surface receptors, with additional uptake via HSPG and LRP. Pronase treatment reduced uptake of 6:4 TGRP, indicating that non-canonical, protein-mediated mechanisms also contribute. This dual uptake likely enhances cellular uptake and accelerates blood clearance [76] (Figure 4). In mice, we confirmed these findings in the liver and peripheral tissues. The liver, the primary organ for TGRP clearance, showed no significant uptake differences with 6:4 or 2:8 TGRP after lactoferrin or apoE treatment. However, extrahepatic tissues, such as muscle, exhibited increased uptake, particularly with 6:4 and 2:8 emulsions. It has been suggested that the differences in uptake may be attributed to the various TGRP particle sizes and hydrolysis rates in cells. Our MCT and *n*-3 TGRP in this study both had smaller particle sizes (Table 1), likely promoting faster lipolysis [26]. Consistent with prior findings, including those by Lutz et al. [82], smaller TGRP particle size reduces LpL and hepatic lipase activity but enhances blood TG clearance, especially for MCT, which hydrolyze quickly due to their greater solubility with shorter acyl tails. Rapid hydrolysis of MCT accelerates the release of MC FA from MCT TGRP and the formation of remnant particles. Our preliminary studies showed that lipolysis rates increased with MCT content, independent of total TG levels. Previous nuclear magnetic resonance (NMR) studies have shown that inclusion of MCT in TGRP alters the phospholipid bilayer organization of model membranes and increases phospholipid mobility, resulting in “leakier” and more “fluid” membranes that, as we hypothesize, can promote *n*-3 FA cellular enrichment [83,84,85,86]. Furthermore, MCT modulates membrane phospholipid bilayer organization by excluding other TGs, such as LCT, from the particle surface [83]. Such changes may explain the inefficient lipolysis of *n*-3 LCT in mixed MCT/LCT emulsions [83] and support our hypotheses that most released FA are derived from MCT from mixed MCT/*n*-3 TGRP. Previous reports found MCT:FO 8:2 TGRP to be more efficient in increasing hydrolysis of MCT compared to LCT and MCT:LCT:FO 5:4:1 TGRP [35]. When MCTs were combined with *n*-3 in TGRP in this study, more TGRP uptake occurred in cells for all three mixed emulsions than the pure forms, and the uptakes of MCT:*n*-3 6:4 and 2:8 emulsions appeared to be the greatest (Figure 1). Both 6:4 and 2:8 TGRP contained higher levels of *n*-3 TG. Their higher *n*-3 TG content may slow lipolysis and preserve particle integrity during uptake. Indeed, we showed in vitro that inefficient hydrolysis of pure FO emulsion by LpL results in limited release of EPA and DHA [26]. A small amount of MCT may enhance membrane solubility and flexibility, facilitating the remaining *n*-3 incorporation. Further studies are needed to explore how *n*-3 TG particle size influences uptake and to examine the role of FA transporters like CD36 in mediating lipolysis product specificity.

We tested the blood clearance of various TGRPs in an LpL-KO mouse model that was rescued by expressing LpL in muscle to preserve partial LpL lipolytic activity in blood. Our results show that blood elimination rates and FCR did not differ after injections of various TGRPs, including LCT, pure *n*-3, or mixed MCT:FO 8:2 in the LpL-knockout animal model compared to the wild type. However, the absence of LpL in major metabolic tissues, including the liver, markedly decreased cellular lipid uptake, regardless of the differences in the core TG composition of each TGRP. In fact, the heparin-mediated increase in lipolytic rates enhanced FCR and tissue uptake of each tested TGRP, likely including TGRP-derived free FA and remnant particles. Further studies are needed to uncover the underlying enrichment mechanisms and determine specific ratios of MCT to *n*-3 TG to optimize the delivery of *n*-3 to target cells. Assessing whether *n*-3 enrichment depends on specific *n*-3 DHA or EPA or the inclusion of MCT will provide direct evidence of the correlation between *n*-3 internalization and changes in FA composition and specific lipid species in cells and membranes.

When analyzing metabolic pathways, we found that TGRP, with a higher MCT to *n*-3 TG ratio, particularly 8:2, significantly enhanced both oxidative phosphorylation and glycolytic capacity in both resident and LPS-stimulated macrophages compared to pure MCT or *n*-3 TG alone (Figure 7 and Figure 8). MCT:*n*-3 8:2 also demonstrates anti-inflammatory effects (Figure 8E and Figure 9), supporting our hypothesis of a synergistic effect of combining MCT with *n*-3. MCT-containing emulsions are rapidly oxidized through CPT-independent mechanisms, potentially promoting a metabolic shift from an M1- to an M2-like macrophage phenotype, which requires increased mitochondrial respiration during stress [43]. Interestingly, as the MCT:*n*-3 ratio decreases, the metabolic benefits diminish. Pure *n*-3 TGRP notably suppressed mitochondrial respiration and glycolytic function (Figure 5 and Figure 7), which may delay or prevent ROS production. Nevertheless, *n*-3 TGRP still reduced pro-inflammatory cytokine expression in a dose-dependent manner in vitro (Figure 8E), and both MCT and *n*-3 TGRP had differentially lowered systemic markers such as CRP and IL-6 in vivo (Figure 9). By contrast, LCT showed no protective effects in mice exposed to acute LPS. Prior studies have reported that MCT can decrease M1 markers (CD86, MHC-II) and increase M2 markers in macrophages treated with pure oil [39], exhibiting anti-inflammatory action. Additionally, elevated IL-6 and CRP levels are known to suppress hepatic glycolysis and contribute to fatty liver disease progression [87]. Similarly, CRP induces decreased glycolysis in hepatic cells [88]. Our data suggest that acute treatment with MCT or MCT:*n*-3 8:2 may counter these effects by preserving mitochondrial and glycolytic activity (Figure 8), potentially protecting against metabolic dysfunction under inflammatory stress. Our data demonstrate that MCT:*n*-3 TGRP, particularly at an 8:2 ratio, most effectively reduced inflammatory markers CRP and IL-6 across plasma, liver, and spleen following LPS challenge. Compared to pure MCT, *n*-3, or LCT emulsions, the 8:2 formulation consistently achieved greater suppression of both systemic and tissue-specific inflammation, suggesting a synergistic anti-inflammatory effect. These findings support the role of MCT-enhanced *n*-3 lipid emulsions in modulating immune responses, likely through preservation of metabolic function and attenuation of pro-inflammatory signaling in key immune-regulating organs. In contrast, prolonged LCT infusion has been associated with worsened disease outcomes. Understanding how lipid emulsions modulate substrate oxidation in immune cells may clarify their effects on cellular metabolism and inform strategies for immunomodulation under inflammatory conditions. A better understanding of tissue-specific effects would benefit from direct measurements of SPMs, macrophage polarization, and lipidomic profiles. Future research should explore the effects of these emulsions in chronic inflammatory conditions like metabolic syndrome or fatty liver disease, where immune dysregulation and tissue remodeling occur.

Another potential mechanism underlying the anti-inflammatory properties of *n*-3 FA is the regulation of levels and activity of intracellular lipid signaling molecules. We have observed a reduction in several major lipid species, especially those that are key in mediating pro-inflammatory responses, such as ceramides and diglycerides mediated by increasing *n*-3 content after 4 h incubation in whole cell lysates (Figure 1). Other studies have observed that, similarly, DHA ethyl esters from *n*-3 supplementation replace linoleic acid in mitochondrial lipidomes and ultimately affect respiratory enzyme levels. Our cell-model study highlights this, as we observed a significant increase in complex IV activity after *n*-3 TGRP in macrophages, likely reducing ROS production in the inflammatory response. MCT:*n*-3 TGRP, particularly 8:2, appeared to increase complex I activity and thus enhance oxygen respiration as compared to *n*-3 TGRP without MCT (Figure 6). The results may represent related structural changes in membrane microstructure and the activity of many cellular membrane-associated physiological pathways in inflammation. LPS has been shown in a number of studies to increase the expression of mitochondrial membrane complexes I and IV of the electron transport chain, thereby upregulating mitochondrial respiration and mitochondrial biogenesis [49]. It has also been shown to reverse electron flux through complex III, driving ROS production. Given the increased oxidative stress and evidence of mitochondrial dysfunction in inflammation, biogenesis and mitophagy may coordinate to maintain ATP production within the viable range, upregulating glycolysis for energy needs [49,89]. The effects of MCT:*n*-3 TGRP on mediating changes in the context of *n*-3-related bioactive metabolites will be evaluated in future experimental cell models. Nonetheless, measuring cellular concentrations of major oxygenated catabolites and production of lipid metabolites and mediators will provide more definitive answers on the interaction of the proposed MCT:*n*-3 TGRPs and the membrane lipid architecture and its effects on the subsequent biological activity.

While our study provides important mechanistic insights into the metabolic and anti-inflammatory effects of MCT:*n*-3 emulsions, it is important to acknowledge the limitations inherent in using cell culture and animal models. These systems, while valuable for controlled investigation of acute inflammatory responses, may not fully replicate the complexity of human conditions such as sepsis, including organ-specific interactions, immune heterogeneity, and long-term physiological adaptations [49]. Although our study utilized LPS as an acute inflammatory stimulus, which models aspects of systemic inflammation, it is noted that it does not fully recapitulate the complexity of clinical sepsis. Notably, DHA has been shown to modulate mitochondrial complex activity by altering lipid composition and cristae structure [42,90,91], while EPA has been reported to shift mitochondrial metabolism toward glucose oxidation in septic models [92]. These observations are consistent with our findings that *n*-3 FA can exert distinct bioenergetic effects. While our study primarily examined acute inflammatory responses, the rapid enrichment of cell membranes and organs with *n*-3 FA—particularly following administration of MCT:FO (8:2) emulsions—suggests a potential mechanism for early metabolic support. This early uptake may help stabilize immune cell bioenergetics and facilitate inflammation resolution, which could have downstream benefits in preventing sustained immune activation or metabolic dysfunction. These results highlight the therapeutic relevance of uptake kinetics and emulsion composition, with implications for both acute intervention and longer-term immunometabolic resilience. Future studies in humanized or clinically relevant models will be essential to validate and extend the translational potential of these findings.

## 5. Conclusions

Our studies systematically compared the metabolic and anti-inflammatory effects of TGRP composed of varying MCT:*n*-3 TG ratios in both in vitro and in vivo models. We demonstrate, for the first time, that the 8:2 MCT:*n*-3 TGRP optimally preserves mitochondrial respiration and glycolytic capacity in macrophages while reducing inflammatory markers in plasma and key metabolic organs. These findings reveal a synergistic interaction between MCT and *n*-3 that is ratio-dependent, providing a novel and rational basis for the development of optimized lipid emulsions in acute inflammatory conditions.

## Figures and Tables

**Table 1:** Characterization of TGRP particle size and electronic dispersity (zeta (z)-potential).

TGRP	MCT	MCT:*n*-3 8:2	MCT:*n*-3 6:4	MCT:*n*-3 2:8	*n*-3
(10%TG by wt)					
Size (d.nm)	191.6 ± 2.78	198.8 ± 3.63	198.13 ± 7.49	205.7 ± 5.65	182.93 ± 4.76
Ζ-Potential (mV)	−12.13 ± 0.83	−26.4 ± 8.20	−25.6 ± 5.03	−31.93 ± 1.86	−30.13 ± 8.34

## Data Availability

The original contributions presented in this study are included in the article/Supplementary Material. Further inquiries can be directed to the corresponding author(s).

## Supplementary Materials

The following supporting information can be downloaded at: https://www.mdpi.com/article/10.3390/nu17111889/s1, Figure S1: Characterization of mouse immune responses to LPS challenge. (A) C57BL/6J mice were IP injected with various doses of LPS. Blood leukocyte subsets – neutrophils (Neu) and monocytes (Mon) were measured. (B) Plasma CRP levels were measured 24 h post-injection by ELISA. (C) Time course of plasma CRP and IL-6 levels after LPS injection, n = 3-4. Data were expressed as Mean ± SEM, n = 3-5, * *p* < 0.05, ** *p* < 0.01, *** *p* < 0.001, **** *p* < 0.0001. One-way ANOVA with Tukey’s multiple comparisons. Figure S2: MCT/n-3 2:8 TGRP increase cellular TG utilization in immune cells. J774 macrophage cells were incubated with [^3^H]CE-labeled pure MCT, n-3 TGRP, or TGRP in which n-3 TG was mixed with MCT (MCT:n-3 8:2, MCT:n-3 6:4, MCT:n-3 2:8) at 37 °C for four h (200 µg TG/mL). Cellular TG utilization is calculated as the TG uptake/TG mass ratio based on our previous reports. Data is expressed as the mean ± SEM (n=3 or 4) and denoted as * *p* < 0.05 utilizing one-way ANOVA followed by Tukey’s multiple comparisons. Figure S3: Non-hepatic organ uptake of various TGRP in mice. C57BL/6J mice were intravenously injected with [^3^H]CE-labeled emulsions (0.016 mg TG/g BW) in the absence or presence of lactoferrin (0.07 mg/g BW), apoE, or heparin (0.4 units/g BW) injected 1 min before TGRP injection. (A) Organ uptake in the heart, spleen, muscle, and adipose were measured in the absence or presence of (A) lactoferrin and apoE or (B) heparin. Data were expressed as Mean ± SEM, n = 3-5, * *p* < 0.05, ** *p* < 0.01, *** *p* < 0.001, **** *p* < 0.0001, and compared with NT group by 2-way ANOVA with Dunnett’s multiple comparisons. Figure S4: Characterization of Inflammatory cytokine mRNA levels in macrophages. J774A2 macrophages were stimulated with LPS at different concentrations (0.01–1 µg/mL) after 16 or 24-h incubation. The mRNA expression levels of cytokines TNF-α (A), IL-6 (B), and IL-1β (C) were analyzed by quantitative real-time PCR. Data expressed as mean ± SEM.

## Abbreviations

DHA (docosahexaenoic acid); EPA (eicosapentaenoic acid); FA (fatty acid); FCR (fractional catabolic rate); FFA (free fatty acid); FO (fish oil); LpL (lipoprotein lipase); LPS (lipopolysaccharide); MCT (medium-chain triglyceride); *n*-3 (omega-3); TG (triglyceride); TGRP: triglyceride-rich emulsion particle.
