# Supplementary material for: Exploring the Synergistic Action of Medium-Chain Triglycerides and Omega-3 Fatty Acids to Enhance Cellular Uptake and Anti-Inflammatory Responses
Source: Nutrients. 2025 May 31;17(11):1889. doi: 10.3390/nu17111889 (PMC12158061; doi:10.3390/nu17111889)
Supplement: Supplementary file 1 [file nutrients-17-01889-s001.zip › nutrients-3656893-supplementary.pdf]

Supplemental Figures

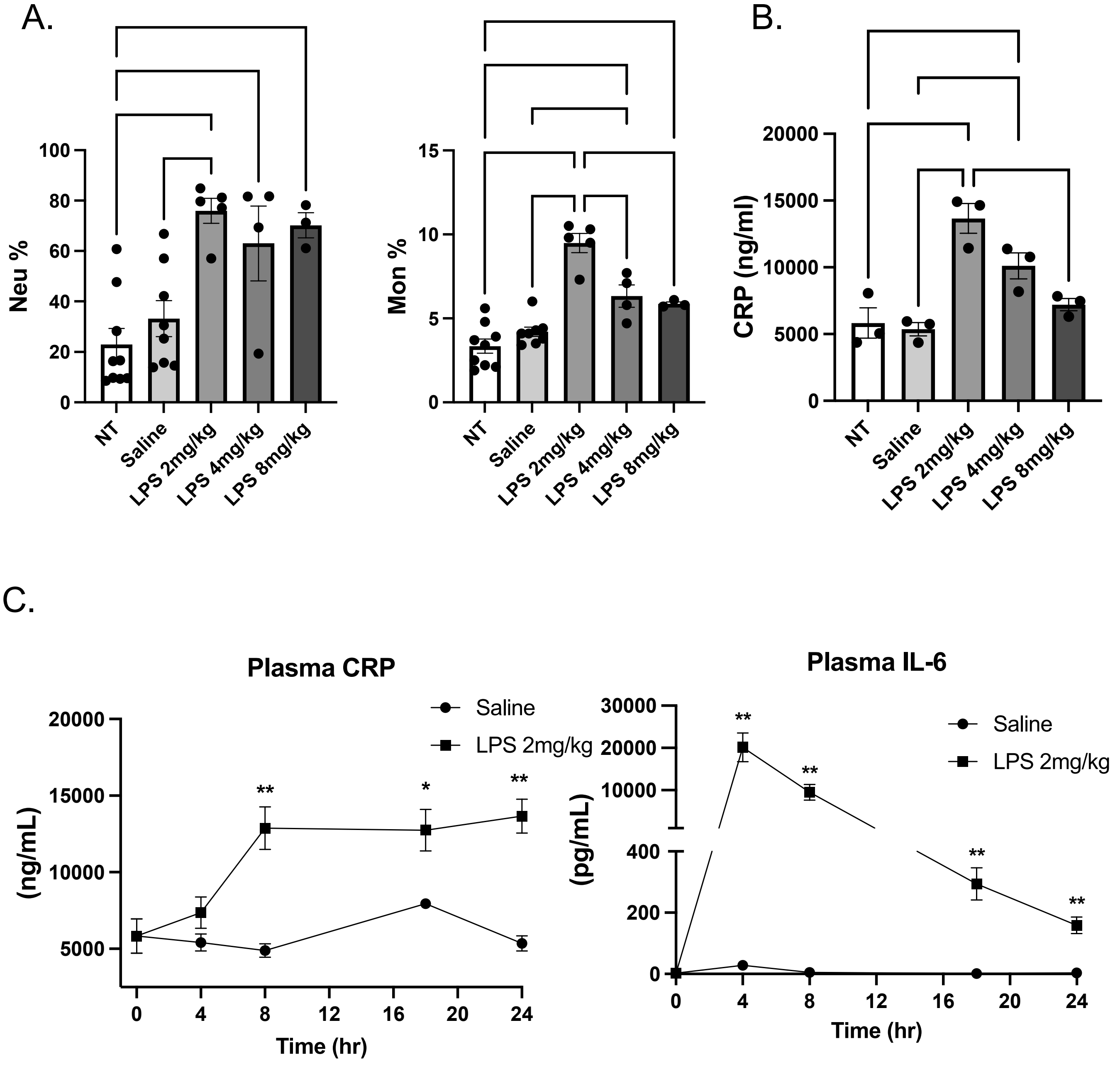

Supplemental Figure S1:Characterization of mouse immune responses to LPS challenge. (A) C57BL/6J mice were IP injected with various doses of LPS. Blood leukocyte subsets–neutrophils (Neu) and monocytes (Mon) were measured. (B) Plasma CRP levels were measured 24 h post-injection by ELISA. (C) Time course of plasma CRP and IL-6 levels after LPS injection, n=3-4. Data were expressed as Mean ± SEM, n = 3-5, * $p < 0.05$, ** $p < 0.01$, *** $p < 0.001$, **** $p < 0.0001$. One-way ANOVA with Tukey's multiple comparisons.

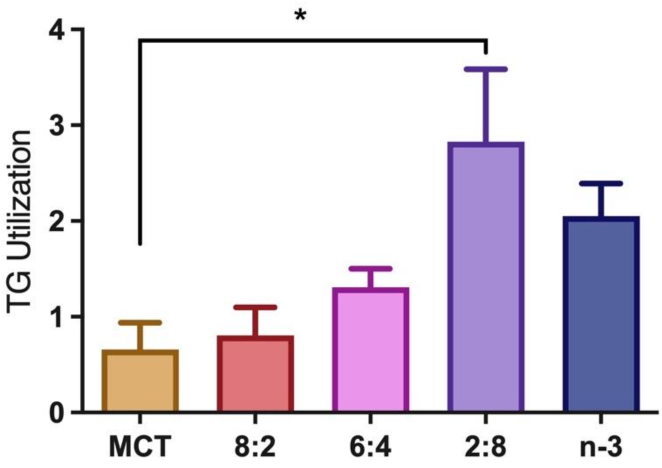

Supplemental Figure S2: MCT/n-3 2:8 TGRP increase cellular TG utilization in immune cells. J774 macrophage cells were incubated with [3H]CE-labeled pure MCT, n-3 TGRP, or TGRP in which n-3 TG was mixed with MCT (MCT:n-3 8:2, MCT:n-3 6:4, MCT:n-3 2:8) at 37ºC for four h (200 µg TG /ml). Cellular TG utilization is calculated as the TG uptake/TG mass ratio based on our previous reports. Data is expressed as the mean ± SEM (n=3 or 4) and denoted as * $p < 0.05$ utilizing one-way ANOVA followed by Tukey's multiple comparisons.

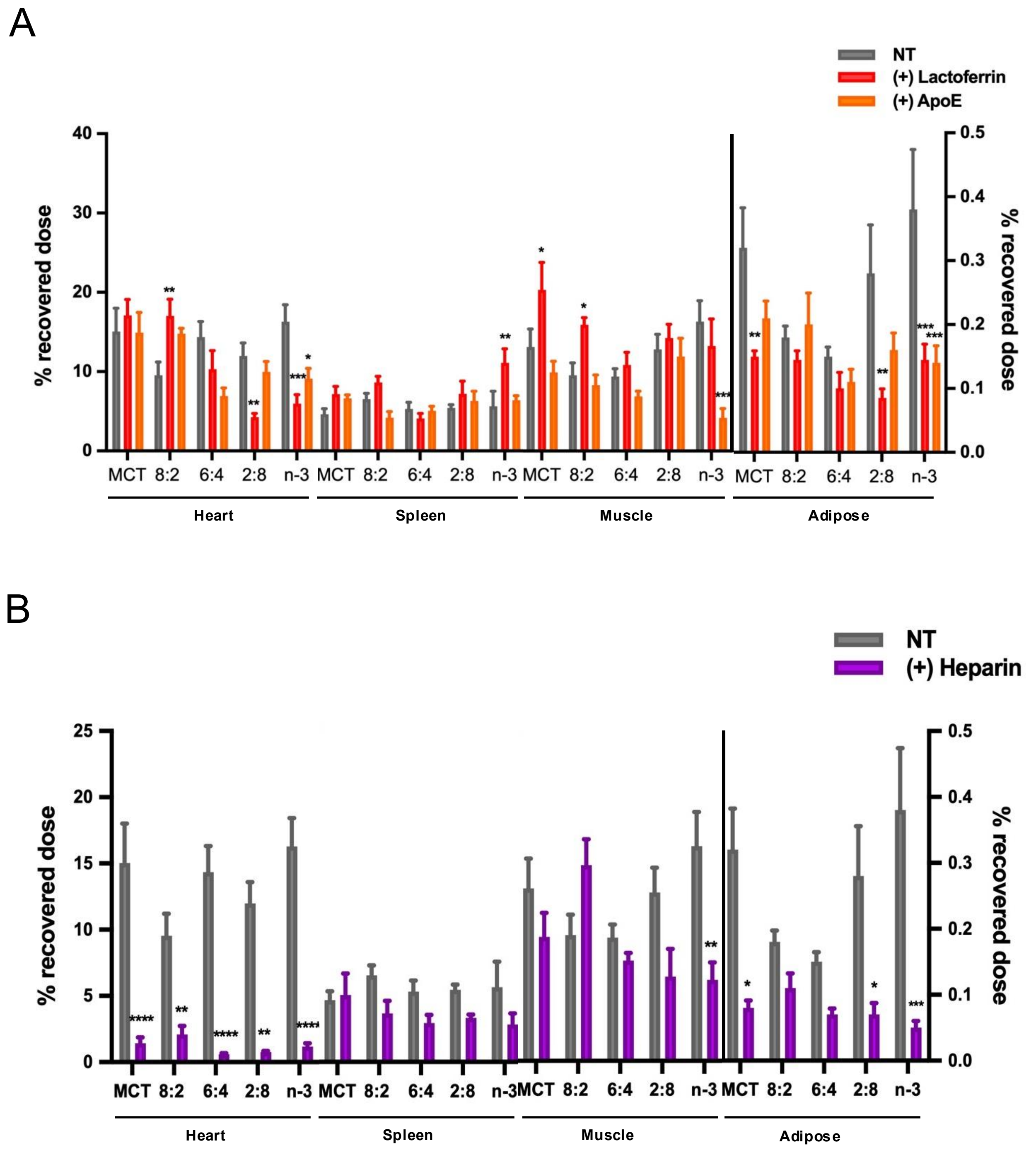

Supplemental Figure S3: Non-hepatic organ uptake of various TGRP in mice. C57BL/6J mice were intravenously injected with [^{3}H]CE-labeled emulsions (0.016 mg TG/g BW) in the absence or presence of lactoferrin (0.07mg/g BW), apoE, or heparin (0.4 units/g BW) injected 1 min before TGRP injection. (A) Organ uptake in the heart, spleen, muscle, and adipose were measured in the absence or presence of (A) lactoferrin and apoE or (B) heparin. Data were expressed as Mean ± SEM, n = 3-5, * $p < 0.05$, ** $p < 0.01$, *** $p < 0.001$, **** $p < 0.0001$, and compared with NT group by 2-way ANOVA with Dunnett's multiple comparisons.

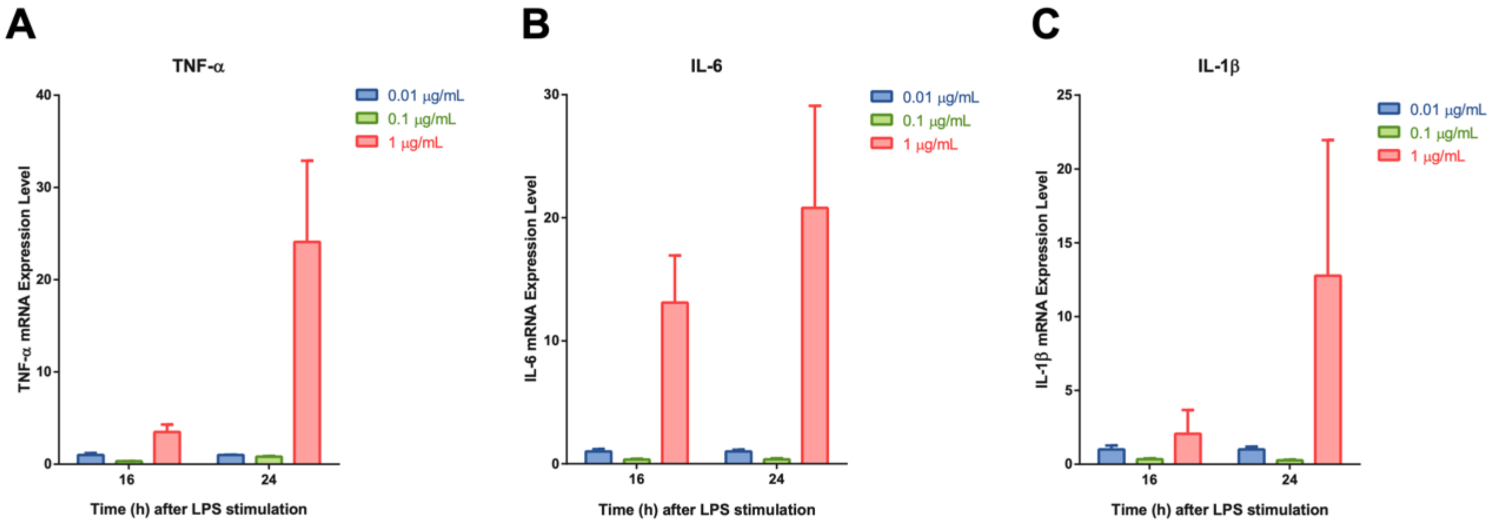

Supplemental Figure S4: Characterization of Inflammatory cytokine mRNA levels in macrophages. J774A2 macrophages were stimulated with LPS at different concentrations (0.01 – 1 μg/mL) after 16 or 24-hour incubation. The mRNA expression levels of cytokines TNF-α **(A)**, IL-6 **(B)**, and IL-1β **(C)** were analyzed by quantitative real-time PCR. Data expressed as mean ± SEM.
